# Evaluation of the smoking cessation effects of QuitAction, a smartphone WeChat platform

**DOI:** 10.18332/tid/161257

**Published:** 2023-04-11

**Authors:** Jianghua H. Xie, Yanfang F. Qiu, Lei Zhu, Yina Hu, Xiaochang Chang, Wei Wang, Lemeng M. Zhang, Ouying Y. Chen, Xianmin Zhong, Xinhua Yu, Yanhui Zou, Rui Zhong

**Affiliations:** 1 Hunan Cancer Hospital/The Affiliated Cancer Hospital of Xiangya School of Medicine, Central South University, Changsha City, China; 2 School of Nursing, Hunan University of Chinese Medicine, China; 3 Department of Otorhinolaryngology Head and Neck Surgery, Xiangya Hospital, Central South University, China; 4 School of Nursing and Health Management, Wuhan Donghu University, Wuhan, China

**Keywords:** mobile medical treatment, WeChat mini program, smoking cessation, evaluation

## Abstract

**INTRODUCTION:**

Many smokers in China desire to quit, though the success rate among adults is low. This study evaluated the effects of QuitAction, a WeChat smoking cessation platform, summarized the intervention experience of the smoking cessation platform, identified aspects of the platform that necessitated improvement, and provided references for further optimization of the smoking cessation platform.

**METHODS:**

This single-arm study was conducted in Hunan, China, from September 2020 to October 2021. Regular smokers, who were aged ≥15 years and willing to quit smoking using QuitAction, were recruited. An in-application questionnaire evaluated participants’ baseline smoking status and intention to quit smoking. The QuitAction program included questionnaires regarding the participants’ ongoing smoking cessation status at 24 hours, one week, one month and three months after quitting. The smoking cessation procedure was discontinued if the participant had no intention of continuing. The smoking cessation rate, influencing success factors, frequency of use satisfaction, and helpfulness of QuitAction were recorded.

**RESULTS:**

A total of 303 participants registered and logged into the QuitAction program, including 59 with incomplete information and 64 with no intention of quitting. The study finally included 180 participants. The smoking cessation rate was 33.9% at 24 hours, 27.2% at one week, 26.1% at one month, and 25.0% at three months. QuitAction was reported as helpful by 94.9% of participants and 95.7% were satisfied with the program. Participants with a quitting difficulty score of 80–100 were less likely to quit smoking than participants with a difficulty score of 0–60 (OR=0.28; 95% CI: 0.10–0.78; p=0.015). Participants using the platform ≥5 times were more likely to quit smoking than those who used the platform <5 times (OR=3.59; 95% CI: 1.51–8.52; p=0.004).

**CONCLUSIONS:**

The QuitAction platform provides smoking cessation services that can improve smokers’ success rate and improve user experience satisfaction.

## INTRODUCTION

The tobacco epidemic is one of the most concerning global public health problems. Globally, more than 8 million people die from smoking-related diseases annually^1^. The annual medical expenditure and productivity burden caused by smoking-related diseases in the United States have been reported as $1436 billion^2^. In China, the tobacco epidemic is a serious concern. According to the 2018 China Adult Tobacco Survey report, approximately 308 million adults in China smoke, the largest number in any country^3^. The overall smoking rate in China was 26.6%, significantly higher than the global smoking rate of 19.2%^3^. The smoking rate among men was 50.5% (global smoking rate among men: 36.1%)^4^. However, in 2018, successful smoking cessation rate in Chinese adults was 20.1%, and 19.8% of smokers reported unsuccessful attempts at quitting, including 90.1% who reported not using any smoking cessation method during the quitting process^3^. These findings suggest that smokers are not very aware of smoking cessation methods in China. In addition, approximately 50 million (16.1%) smokers in China intend to quit within one year^3^, suggesting a huge demand for smoking cessation services in China.

The development of mobile healthcare and the popularity of smartphone applications has led to the implementation of mobile smoking cessation platforms. These mobile smoking cessation platforms are effective and eliminate the time and space limitations of outpatient smoking cessation services. In addition, the platforms are inexpensive, easy to use, and can be used by many users simultaneously^5,6^. These include SmartQuit, SmokeFree28, and Q Sense^7^. The content and style of the applications are continuously updated, and the service objectives and scope are often expanded.

WeChat was named ‘the world’s leading internet scientific and technological achievement’ in 2018. The 47th Statistical Report on the Development of the Internet in China in 2021 indicated that the number of mobile Internet users in China was 986 million, and the number of active WeChat accounts was 1.225 billion in December 2020. Therefore, WeChat mini-programs were available to a large patient population^8^. In June 2021, the number of WeChat mini-programs exceeded 4.3 million, with more than 410 million daily users and an average daily usage time of 22.5 minutes^9^. WeChat mini-programs spanned several industries including living services, videos, online shopping, tourism, and medical care, and covered all aspects of public life^10^. All major hospitals in China use mobile programs for electronic appointment registration and payment, due to the advantage of these programs in terms of resources and time^10^. These programs reduce patients’ waiting time and improve the hospitals’ efficiency. Ye et al.^11^ developed a WeChat mini-program regarding home fall-prevention for urban elderly participants, which improved the understanding of home fall-prevention methods in this population. Our research team surveyed the existing WeChat smoking cessation mini-programs in the Chinese market at the early stage^12^. We found a total of 72 mini-programs related to smoking cessation (up to 16 August 2019), of which only 51 were designed to help smokers quit. These mini-programs offered insufficient support on smoking cessation guidelines and needed more professional, comprehensive tobacco use assessment, and medical staff help.

QuitAction, a WeChat smoking cessation mini-program, was developed by our team. This mini-program can be obtained directly from WeChat and requires no downloading or installation, which is more convenient and provides a better user-experience. QuitAction was developed using the Chinese Clinical Guidelines for Smoking Cessation (2015 edition)^13^. The intervention model of QuitAction was constructed according to the 5As (ask, advise, assess, assist, and arrange) and 5Rs (relevance, risks, rewards, roadblocks, and repetition) methods^13^, and the recommended assessment and follow-up were conducted during smoking cessation. Online medical staff consultation and offline telephone follow-up channels were developed to support smokers during smoking cessation.

This study evaluated the seven-day smoking cessation rates at one month and three months after the QuitAction intervention, the influencing success factors and frequency of use satisfaction; it also summarized the intervention experience of the smoking cessation platform, identified the aspects of the platform that required improvement, and provided references for further optimization of the smoking cessation platform.

## METHODS

### Study design and participants

This single-arm study was conducted in Hunan, China, from September 2020 to October 2021. The QR code for QuitAction was promoted through posters, websites, WeChat, and the public WeChat account of a tertiary hospital.

*Inclusion criteria*

Regular smokers^14^ (smoked at least one cigarette/day for at least six consecutive or cumulative months) who were aged ≥15 years and willing to quit smoking using QuitAction were recruited. All participants possessed a smartphone, could use the smartphone independently, had intentions of quitting smoking, and planned to quit smoking within 30 days.

*Exclusion criteria*

Participants with life-threatening medical conditions and cognitive impairment were excluded from the study.

### Theoretical framework and intervention process

‘Quit Smoking Action’ (QuitAction), the WeChat smoking cessation mini-program was developed following the Chinese Clinical Guidelines for Smoking Cessation (2015 edition)^13^ and uses the 5As and 5Rs methods as its theoretical framework for psychological counselling and support^15^. Specific modes of intervention were provided based on the smokers’ stage of smoking cessation. The 5As method was used to help smokers quit, and the 5Rs method was used to increase the smokers’ motivation and actions to quit.

### Sample size calculation

This study adopted the sample size formula required for a cohort study. According to the preliminary experiment in the early stage of this study, 50% of the smokers in the successful smoking cessation group used the WeChat smoking cessation mini-program ≥5 times, and 20% of the smokers in the unsuccessful group used the WeChat smoking cessation mini-program for <5 times, so *p*_1_=0.5, *p*_0_=0.2, *α*=0.05, *β*=0.10, which gave n=51. Considering the loss of follow-up rate of 20%, the total sample size was ≥122 cases.

### Intervention measures and QuitAction modules

QuitAction includes an automatic guide to quitting smoking, allowing participants to use the platform without assistance from trained healthcare workers. However, the platform automatically notified trained healthcare workers when participants needed to follow the provided guide. The participants completed questionnaires on the QuitAction platform at 24 hours, one week, one month, and three months after their quit date. The platform automatically recorded the responses. If the participants did not complete the questionnaires, healthcare workers were notified by QuitAction. Each participant who did not complete a questionnaire within one day was contacted by telephone. Participants who expressed no interest in quitting smoking were encouraged to discontinue the QuitAction program.

**Figure 1:**
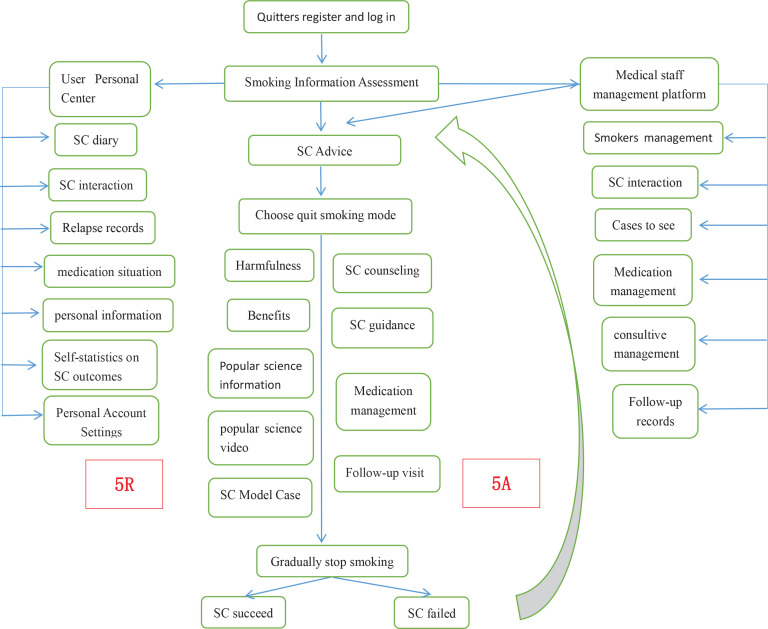
Flowchart of smoking cessation intervention with the QuitAction mini-program

The QuitAction smoking cessation program consists of registration and login, assessment, expert opinion, development of a quit date, consultation methods, follow-up questionnaires, health education, and a personal center. The registration and login allow participants to enter the platform’s main page by scanning the QR code on the recruitment and promotion announcements. The login dialogue box automatically loads. Participants can register and log in using their WeChat identification after authorizing their mobile phone number. Then, participants provide general and smoking-related information and complete the nicotine dependence assessment form. The results are evaluated by smoking cessation experts, who provide advice regarding smoking cessation. The participants are then asked to choose their quit date using three options: quit smoking immediately, stop smoking gradually, or quit smoking at a predetermined date. The participants provide their reasons for quitting smoking. Each step of smoking cessation is detailed according to the smoking cessation guide. QuitAction includes multiple types of consultation, including medical staff with knowledge regarding smoking cessation, nutrition, exercise, psychology, and smoking cessation medications. Medication information included where to buy the medicine, how to take the medication, and adverse reactions. The QuitAction platform did not prescribe or provide drugs directly. At a given follow-up time point, automatic follow-up questionnaires were provided within the platform. Based on the participants’ responses, their smoking cessation status was determined and corresponding guidance was provided. The QuitAction platform included smoking cessation information, videos regarding helpful and harmful habits during smoking cessation, articles, model cases, and other modules to educate the participants regarding the harm caused by tobacco use and to provide motivation for smoking cessation. Last, the personal center of the QuitAction platform includes a quit diary, a message board, a relapse record, personal information, account settings, and quit timing (Figure 1).

### Data collection

The electronic questionnaires and follow-up forms were designed based on the participants’ registration information and a follow-up questionnaire developed by the Tobacco Control Office of the Chinese Center for Disease Control and Prevention^16^. The QuitAction user-experience satisfaction survey and evaluation questionnaire were created for this platform. The questionnaire was divided into the following four parts.

*General questionnaire*

This included collection of information on participant name, sex, age, occupation, and comorbidity information.

*Smoking-related questionnaire*

This collected information on the amount of smoking, smoking age, age of starting smoking, previous attempts at quitting smoking, and the number of smoking cessation attempts. The Chinese version of the Fagerström test for nicotine dependence was used to evaluate the degree of nicotine dependence of the participants^17,18^. The maximum possible score was 10, and patients scoring 0–2 were considered to have very low nicotine dependence. A score of 3–4 was classified as low nicotine dependence, while a score of 5 was regarded as moderate nicotine dependence. A score of ≥6 was considered a heavy nicotine dependence. Quit smoking difficulty score, self-judged how difficult it is to quit smoking, was scored from 0 to 100.

*Simplified follow-up table*

This included smoking status, the number of smokers, and smoking cessation methods, and determined at each follow-up.

*QuitAction user experience satisfaction survey and evaluation questionnaire*

A satisfaction questionnaire was developed specifically for this study. The questionnaire asked participants to evaluate the program’s degree of smoking cessation assistance (categorized as very helpful, helpful, generally helpful, and not helpful). Participant satisfaction with the platform was graded using a 5-point Likert scale (very satisfied, satisfied, generally satisfied, dissatisfied, not satisfied at all). The total satisfaction rate was calculated as the total number of generally satisfied, satisfied and very satisfied responses divided by the total number of responses. The questionnaire also allowed participants to identify the most helpful function of the program and provide suggestions for the improvement of the platform.

### Study outcomes

The primary outcomes of this study were the seven-day smoking cessation rates at one month and three months after the QuitAction intervention. The seven-day smoking cessation rate was defined as the participants’ self-reported abstinence from smoking for the previous seven days at each follow-up. Patients lost to follow-up were considered not to have succeeded at quitting smoking. We analyzed participants’ smoking cessation status and characteristics at follow-up at 3 months, and predicted the influencing factors of successful smoking cessation.

The secondary outcome indicators included the participants’ frequency of using the QuitAction platform and their satisfaction with it. The helpfulness of QuitAction for smoking cessation was also evaluated.

### Statistical analysis

Categorical data are expressed as frequency and percentage. The chi-squared test, Fisher’s exact probability method, or Monte Carlo direct calculation method were used to compare categorical variables. Continuous data with a normal distribution are presented as mean and standard deviation, while those without a normal distribution are presented as median and interquartile range. The normality was determined using the Shapiro-Wilk test. A binary regression analysis was used to identify the factors influencing smoking cessation. Odds ratios (ORs) and 95% confidence intervals (CIs) were evaluated in multivariable analysis. All analyses were conducted using SPSS v. 25.0 (IBM, Chicago). Statistical significance was set at p<0.05.

## RESULTS

### Participant characteristics

A total of 303 persons registered and logged into the QuitAction program from 1 September 2020 to 31 October 2021, including 59 with incomplete information and 64 with no intention to quit smoking. The study finally included 180 participants.

The average participant age was 36.2 ± 12.4 years, and most were male (96.1%, n=173). Nearly half of the participants (48.9%) had a college degree or higher, and 72.2% were urban residents. Approximately one quarter (26.7%) of participants worked in enterprises/commercial/service industries, while 16.1% worked in government/public institutions. The average monthly household income was <3000 RMB (1000 Chinese Renminbi about US$150) in 41.1% of participants, and 16.1% earned >10000 RMB per month. Most participants (60.6%) had a normal body mass index, 27.8% had pre-obesity, and 5.0% were obese. Chronic diseases were reported by 17.2% of participants.

### Participant smoking status

The average years of smoking by the participants was 16.9 ± 11.2 (range: 0.9–46), and 58 (32.2%) participants reported a smoking history of >20 years. The average number of cigarettes smoked per day was 19.7 ± 10.4, and 48 (26.6%) participants smoked more than 20 cigarettes per day. Seventy-eight (43.3%) participants were highly dependent on nicotine, and 32 (17.8%) were moderately dependent. A total of 116 participants (64.4%) reported previous attempts at quitting smoking, and 44.4% reported 1–3 previous attempts. The difficulty of smoking was rated as 0–60 by 36.1% of participants, 61–80 by 31.1%, and 81–100 by 32.8% of participants. Most participants (63.3%) chose to quit smoking due to concerns regarding their health and their family’s health. In comparison, the surrounding environment influenced 20%, and 16.7% decided to quit due to their comorbidities.

### Factors affecting smoking cessation

**Figure 2:**
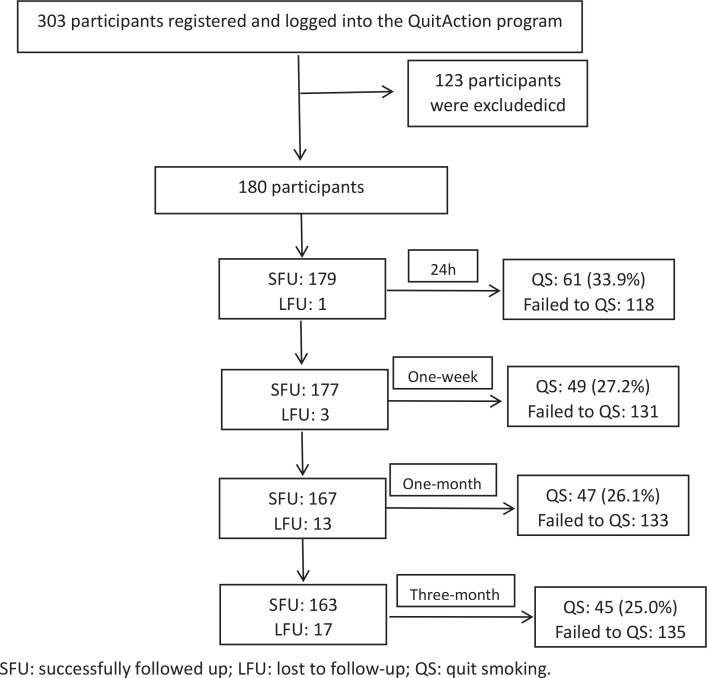
Flowchart for follow-up and successful smoking cessation at each time point

A total of 179 participants completed the 24-hour questionnaire, and the reported smoking cessation rate was 33.9% (Figure 2). In total, 177 participants completed the questionnaire at the follow-up at 1 week, and the reported smoking cessation rate was 27.2%; 167 participants completed the questionnaire at the follow-up at 1 month and at 3 months, and the reported seven-day smoking cessation rate was 26.1% and 25.0%, respectively.

The participants were divided into a successful smoking cessation group (n=45) and an unsuccessful cessation group (n=135) based on their smoking cessation status reported on the questionnaire at follow-up at three months. There were no significant differences in baseline characteristics between the two groups (Table 1). However, the quitting difficulty score (p=0.010) and the frequency of using QuitAction (p=0.005) were significantly different between the two groups. The difficulty of quitting score was rated as 0–60 points by 53.3% of the successful quitters and >60 points by 63.9% of the unsuccessful quitters. Also, 51.1% of the successful group and only 28.1% of the unsuccessful group used the QuitAction program >5 times, respectively (Table 2).

**Table 1:** Clinical characteristics of participants between successful group and unsuccessful group in smoking cessation for 3 months (N=180)^a^

*Variables*	*Total (N=180)*	*Unsuccessful (N=135)*	*Successful (N=45)*	*χ^2^*	*p*
	*n (%)*	*n (%)*	*n (%)*		
**Gender**				0.050	0.824^b^
Male	7 (3.9)	6 (4.4)	1 (2.2)		
Female	173 (96.1)	129 (95.6)	44 (97.8)		
**Residence**				0.332	0.564
Rural	50 (27.8)	39 (28.9)	11 (24.4)		
Urban	130 (72.2)	96 (71.1)	34 (75.6)		
**Age** (years)				4.661	0.324
15–24	36 (20.0)	31 (23.0)	5 (11.1)		
25–34	53 (29.4)	41 (30.3)	12 (26.7)		
35–44	44 (24.4)	29 (21.5)	15 (33.3)		
45–54	26 (14.5)	19 (14.1)	7 (15.6)		
55–64	21 (11.7)	15 (11.1)	6 (13.3)		
**Education level**				3.122	0.373
Primary school or lower	11 (6.1)	6 (4.4)	5 (11.1)		
Middle school	40 (22.2)	32 (23.7)	8 (17.8)		
High school/secondary specialized school	41 (22.8)	30 (22.3)	11 (24.4)		
College school or higher	88 (48.9)	67 (49.6)	21 (46.7)		
**Occupation**				5.393	0.494
Government/institution staff	29 (16.1)	25 (18.5)	4 (8.9)		
Enterprise/business/services	48 (26.7)	34 (25.2)	14 (31.1)		
Farmer	11 (6.1)	6 (4.4)	5 (11.1)		
Worker	24 (13.3)	17 (12.6)	7 (15.6)		
Freelance work	22 (12.2)	17 (12.6)	5 (11.1)		
Non-employment	19 (10.6)	15 (11.1)	4 (8.9)		
Other	27 (15.0)	21 (15.6)	6 (13.3)		
**Monthly household income** (RMB)				1.642	0.650
<3000	74 (41.1)	52 (38.5)	22 (48.9)		
3000–6000	47 (26.1)	37 (27.4)	10 (22.2)		
6001–10000	30 (16.7)	24 (17.8)	6 (13.3)		
>10000	29 (16.1)	22 (16.3)	7 (15.6)		
**BMI** (kg/m^2^)				-	0.585*
<18.5	12 (6.6)	10 (7.4)	2 (4.4)		
18.5–24.9	109 (60.6)	82 (60.8)	27 (60.0)		
25.0–29.9	50 (27.8)	35 (25.9)	15 (33.3)		
30.0–34.9	9 (5.0)	8 (5.9)	1 (2.3)		
**Underlying disease**				0.117	0.732
No	149 (82.8)	111 (82.2)	38 (84.4)		
Yes	31 (17.2)	24 (17.8)	7 (15.6)		

a A total of 180 participants attended the QuitAction program in Hunan, China, from September 2020 to October 2021.

b Continuous correction value.

* Monte Carlo p value. RMB: 1000 Chinese Renminbi about US$150.

**Table 2:** Smoking and QuitAction program usage characteristics of participants between successful group and unsuccessful group in smoking cessation for 3 months (N=180)^a^

*Characteristics*	*Total (N=180)*	*Unsuccessful (N=135)*	*Successful (N=45)*	*χ^2^*	*p*
	*n (%)*	*n (%)*	*n (%)*		
**Years of smoking**				-	0.584*
0–1	5 (2.8)	4 (3.0)	1 (2.2)		
2–10	58 (32.2)	45 (33.3)	13 (28.9)		
11–20	59 (32.8)	47 (34.8)	12 (26.7)		
21–30	32 (17.8)	22 (16.3)	10 (22.2)		
**Daily cigarette consumption** (sticks)				4.515	0.211
1–10	46 (25.6)	33 (24.4)	13 (28.9)		
11–20	86 (47.8)	66 (48.9)	20 (44.4)		
21–30	28 (15.6)	18 (13.3)	10 (22.2)		
>30	20 (11.0)	18 (13.4)	2 (4.5)		
**Fagerström score**				1.739	0.628
0–2	42 (23.3)	29 (21.5)	13 (28.9)		
3–4	28 (15.6)	23 (17.0)	5 (11.1)		
5	32 (17.8)	25 (18.5)	7 (15.6)		
≥6	78 (43.3)	58 (43.0)	20 (44.4)		
**Previous quit attempts**				0.517	0.472
Yes	116 (64.4)	89 (65.9)	27 (60.0)		
No	64 (35.6)	46 (34.1)	18 (40.0)		
**Previous quit attempts**				1.733	0.420
0	64 (35.6)	46 (34.1)	18 (40.0)		
1–3	80 (44.4)	59 (43.7)	21 (46.7)		
>3	36 (20.0)	30 (22.2)	6 (13.3)		
**Quit smoking difficulty score**				9.142	0.010
0–60	65 (36.1)	41 (30.4)	24 (53.3)		
61–80	56 (31.1)	43 (31.8)	13 (28.9)		
81–100	59 (32.8)	51 (37.8)	8 (17.8)		
**Reasons and motivation for quitting**				7.035	0.071
Decline in health	30 (16.7)	20 (14.9)	10 (22.2)		
Focus on your health	114 (63.3)	89 (65.9)	25 (55.6)		
Affected by the surrounding environment	14 (7.8)	13 (9.6)	1 (2.2)		
Other	22 (12.2)	13 (9.6)	9 (20.0)		
**QuitAction program usage** (times)				7.943	0.005
<5	119 (66.1)	97 (71.9)	22 (48.9)		
≥5	61 (33.9)	38 (28.1)	23 (51.1)		

* Monte Carlo p value.

a A total of 180 participants attended the QuitAction program in Hunan, China, from September 2020 to October 2021.

Combined with univariate analysis results and professional judgment, the following were selected for binary logistic regression analysis: age, education level, years of smoking, daily cigarette consumption, Fagerström score, previous quit attempts, quit smoking difficulty score, and QuitAction program usage times. Participants who reported a difficulty score of 80–100 were less likely to quit than those who reported a difficulty score of 0–60 (OR=0.28; 95% CI: 0.10–0.78; p=0.015). Participants who used the QuitAction program ≥5 times were more likely to quit smoking successfully than those who used it less frequently (OR=3.59; 95% CI: 1.51–8.52; p=0.004) (Table 3).

**Table 3:** Binary logistic regression analysis* for smoking cessation predictive factors

Variable	β	p	OR (95% CI)
**Age** (years)			
15–24 (Ref.)			1
25–34	0.310	0.664	1.36 (0.34–5.51)
35–44	1.256	0.170	3.51 (0.59–21.08)
45–54	-0.235	0.831	0.79 (0.09–6.82)
55–64	-0.538	0.705	0.58 (0.04–9.48)
**Education level**			
Primary school or lower (Ref.)			1
Middle school	-1.558	0.067	0.21 (0.04–1.11)
High school/secondary specialized school	-1.273	0.119	0.28 (0.06–1.39)
College school or higher	-1.032	0.183	0.36 (0.08–1.63)
**Years of smoking**			
0–1 (Ref.)			1
2–10	0.011	0.994	1.01 (0.07–15.68)
11–20	-0.704	0.634	0.50 (0.03–8.94)
21–30	0.605	0.692	1.83 (0.09–36.69)
>30	1.549	0.358	4.71 (0.17–128.04)
**Daily cigarette consumption** (sticks)			
1–10 (Ref.)			1
11–20	-0.096	0.864	0.91 (0.30–2.73)
21–30	0.307	0.675	1.36 (0.32–5.72)
>30	-1.567	0.129	0.21 (0.03–1.58)
**Fagerström score**			
0–2 (Ref.)			1
3–4	-0.241	0.736	0.79 (0.19–3.18)
5	-0.200	0.772	0.82 (0.21–3.17)
≥6	0.074	0.900	1.07 (0.34–3.44)
**Previous quit attempts**			
Yes (Ref.)			1
No	0.206	0.650	1.23 (0.51–2.99)
**Quit smoking difficulty score**			
0–60 (Ref.)			1
61–80	-0.623	0.194	0.54 (0.21–1.37)
81–100	-1.281	0.015	0.28 (0.10–0.78)
**QuitAction program usage** (times)			
<5 (Ref.)			1
≥5	1.279	0.004	3.59 (1.51–8.52)

* Combined with univariate analysis results and professional judgment, the following were selected for binary logistic regression analysis: age, education level, years of smoking, daily cigarette consumption, Fagerström score, previous quit attempts, quit smoking difficulty score, and QuitAction program usage times.

### QuitAction user experience and satisfaction

The average number of times each participant used the QuitAction platform was 6.3 ± 7.6 (range: 2–46), and 33.9% of participants used the platform ≥5 five times. At the last follow-up, 61.2% of participants reported that the health education module was the most helpful, and 55.2% reported that the knowledge related to smoking cessation in the science information helped improve their determination to quit smoking. The follow-up guidance was reported as a very helpful and effective resource by 12.1% of participants; 4.3% of the participants said the smoking cessation program could help them quit smoking according to the established plan. Individual participants felt the program should include an automatic pop-up reminder regarding smoking cessation.

The QuitAction platform was rated as very helpful by 26.4% of participants, helpful by 47.9%, generally helpful by 19.6%, and not helpful by 6.1%. Most participants (50.3%) were satisfied with the program, while 25.8% were very satisfied, 19.6% were generally satisfied, 4.3% were not satisfied, and no participants were very dissatisfied.

## DISCUSSION

Recent studies have reported that mobile applications are useful tools to promote the implementation of desired behaviors, including smoking cessation^19,20^. In this study, the self-reported seven-day smoking cessation rate was 25% after three months of using QuitAction, which is similar to the seven-day smoking cessation rate after six weeks of using QuitGuide (25.0%) in China^21^, and higher than the smoking cessation rate of Chinese smokers when no application or method is used (4%)^22^. The three-month continuous smoking cessation rate of participants who used the Tobbstop mobile application was significantly higher than that of non-users^23^. Bricker et al.^24^ reported that 21% of users of the SmartQuit platform had quit smoking for at least seven days after two months, and 84% of the participants were satisfied with the application. Smoking abstinence using mobile smoking cessation platforms is more likely to continue at one, three, and six months after quitting^20^. In addition, interventions using WeChat to send smoking-related messages regularly are effective in helping smokers quit^25^. Zhang et al.^26^ conducted a 28-day smoking cessation intervention for quitters through telephone follow-up and the WeChat platform. They set up WeChat groups to share smoking cessation information (videos and scientific knowledge). Smoking cessation experts followed up by telephone every two days to give smoking cessation guidance and other measures. The results showed that the participation rate was as high as 95.4% and the 7-day point smoking cessation rate was 65.7% (69/105). The combination of telephone follow-up and a WeChat mini program is feasible for implementing smoking cessation intervention.

The participants’ success in this study may be because of the fact that the QuitAction platform was developed based on the 5As and 5Rs methods adopted in the Chinese Clinical Guidelines on Smoking Cessation. The 5As method was used to construct specific smoking cessation interventions for participants. After assessing the participants’ reported smoking-related information, the QuitAction platform advises participants to quit smoking with clear, strong, and individualized information, provides participants with online smoking cessation counselling and help services, connects participants with medical staff trained in smoking cessation to address their challenges and doubts, and conducts regular online follow-up. The results of this study indicate that the frequency of using the QuitAction platform during smoking cessation was related to the success of smoking cessation, as participants who used the WeChat program ≥5 times were more likely to quit smoking than those who used it less frequently. A previous study reported that fully adherent participants were more than four times more likely to quit smoking than those with incomplete adherence^27^. Another previous study reported^28^ that increasing the amount of behavioral support may increase the success rate of smoking cessation by 10–20%. Therefore, participants who used the QuitAction platform more often may be able to obtain more behavioral and psychosocial support and achieve better smoking cessation outcomes.

The 5Rs method was used to provide participants with smoking cessation recommendations. The health education modules included in the QuitAction module provided smokers with knowledge regarding the harmful effects of smoking and help to quit smoking. By browsing the health information and videos, users of QuitAction can strengthen their desire to quit smoking and gain valuable skills, ultimately increasing their success rate. Text and graphic health warnings can increase the public’s awareness of the potential health hazards of smoking tobacco, thus encouraging smokers to quit smoking^29^. Evans et al.^30^ suggested that pictures with more negative emotional responses increase the risk-perception of smoking and intention to quit in adults and adolescents, more than plain text warnings.

In this study, the difficulty score of quitting smoking was an independent factor related to the success of smoking cessation. The participants who reported a higher difficulty quitting smoking were less likely to quit smoking than those who reported lower difficulty. This finding may be related to the self-efficacy of the participants. Lower difficulty scores of quitting smoking are linked to more confidence, higher self-efficacy, and a higher probability of quitting smoking^31^. Individuals with higher self-efficacy are more likely to try to quit smoking^31^. Rajani et al.^32^ reported that mobile smoking cessation platforms can significantly increase smokers’ self-efficacy and motivation to quit smoking.

### Limitations

This study is not without limitations. First, it was not a randomized controlled trial, and the success of smoking cessation was self-reported with no confirmation using biochemical indicators or exhaled CO. These limitations may explain differences between the smoking cessation rates of the current study and previous studies. The long-term effects of mini-programs regarding smoking cessation should be evaluated and improved via future randomized controlled trials with large sample sizes and long follow-up times.

## CONCLUSIONS

This study evaluated the smoking cessation effects of the QuitAction smoking cessation platform. The platform resulted in satisfactory smoking cessation rates that were higher than those achieved by smokers who tried to quit independently. Most participants in this study reported the program as helpful and had better user-experience satisfaction. Lower difficulty scores of quitting smoking and using the QuitAction platform more often achieved better smoking cessation outcomes. It is feasible to add popular science knowledge and pictures about tobacco hazards to the QuitAction smoking cessation platform, improve smokers’ motivation to quit smoking, and urge them to use the platform and carry out smoking cessation actions. Applying smoking cessation intervention offline or online and combining telephone follow-up were the options for smoking cessation.

## Data Availability

The data supporting this research are available from the authors on reasonable request.
